# Targeted Lipidomic Analysis of Aqueous Humor Reveals Signaling Lipid-Mediated Pathways in Primary Open-Angle Glaucoma

**DOI:** 10.3390/biology10070658

**Published:** 2021-07-13

**Authors:** Nadezhda V. Azbukina, Dmitry V. Chistyakov, Sergei V. Goriainov, Vladislav I. Kotelin, Elena V. Fedoseeva, Sergey Yu. Petrov, Marina G. Sergeeva, Elena N. Iomdina, Evgeni Yu. Zernii

**Affiliations:** 1 Faculty of Bioengineering and Bioinformatics, Moscow Lomonosov State University, 119234 Moscow, Russia; nadezhda.azbukina@gmail.com; 2 Belozersky Institute of Physico-Chemical Biology, Lomonosov Moscow State University, 119992 Moscow, Russia; mg.sergeeva@gmail.com; 3 SREC PFUR, Peoples’ Friendship University of Russia, 117198 Moscow, Russia; goryainovs@list.ru; 4 Helmholtz National Medical Research Center of Eye Diseases, 105062 Moscow, Russia; vikotelin@ya.ru (V.I.K.); dr.elfed23@gmail.com (E.V.F.); glaucomatosis@gmail.com (S.Y.P.); iomdina@mail.ru (E.N.I.)

**Keywords:** primary open-angle glaucoma, aqueous humor, tear fluid, lipidomics, signaling lipids, polyunsaturated fatty acids, oxylipins, phospholipid derivatives

## Abstract

**Simple Summary:**

Analysis of the eye liquids collected from a cohort of primary open-angle glaucoma patients identified signaling lipids, the pattern of which suggests a role of arachidonic acid/platelet activating-factor (PAF)-dependent pathways and oxidative stress in the pathogenesis of the disease and provides novel targets for its diagnostics and treatment.

**Abstract:**

Primary open-angle glaucoma (POAG) is characterized by degeneration of retinal ganglion cells associated with an increase in intraocular pressure (IOP) due to hindered aqueous humor (AH) drainage through the trabecular meshwork and uveoscleral pathway. Polyunsaturated fatty acids and oxylipins are signaling lipids regulating neuroinflammation, neuronal survival and AH outflow. Among them, prostaglandins have been previously implicated in glaucoma and employed for its treatment. This study addressed the role of signaling lipids in glaucoma by determining their changes in AH accompanying IOP growth and progression of the disease. Eye liquids were collected from patients with POAG of different stages and cataract patients without glaucoma. Lipids were identified and quantified by UPLC-MS/MS. The compounds discriminating glaucoma groups were recognized using ANCOVA and PLS-DA statistic approaches and their biosynthetic pathways were predicted by bioinformatics. Among 22 signaling lipids identified in AH, stage/IOP-dependent alterations in glaucoma were provided by a small set of mediators, including 12,13-DiHOME, 9- and 13-HODE/KODE, arachidonic acid and lyso-PAF. These observations correlated with the expression of cytochromes P450 (CYPs) and phospholipases A2 in the ocular tissues. Interestingly, tear fluid exhibited similar lipidomic alterations in POAG. Overall, POAG may involve arachidonic acid/PAF-dependent pathways and oxidative stress as evidenced from an increase in its markers, KODEs and 12,13-DiHOME. The latter is a product of CYPs, one of which, CYP1B1, is known as POAG and primary congenital glaucoma-associated gene. These data provide novel targets for glaucoma treatment. Oxylipin content of tear fluid may have diagnostic value in POAG.

## 1. Introduction

Glaucoma is a highly prevalent neurodegenerative ocular disorder affecting more than 70 million people worldwide. In most cases, it is characterized by a transient or sustained increase in intraocular pressure (IOP) leading to apoptotic death of retinal ganglion cells (RGCs), loss of the retinal nerve fiber layer and excavation of the optic nerve head. In the early stages, the disease is often asymptomatic since defects of the visual field initially develop on its periphery. However, the progression of optical neuropathy in the absence of the appropriate treatment may result in deterioration of the central vision and ultimately blindness [1].

Currently, glaucoma is regarded as a multifactorial disease with a wide range of etiologies and complex pathogenesis. Under normal conditions, IOP is maintained by the delicate balance between secretion of aqueous humor (AH) by the ciliary body and its outflow through the trabecular meshwork (TM) into the Schlemm canal as well as via the uveoscleral pathway. In open-angle glaucoma, the AH outflow is hindered, yielding and increase in IOP up to 20–30 mmHg [2]. Development of primary open-angle glaucoma (POAG) is related to a malfunctioning of the TM and/or aberrations of the structural and biomechanical features of the corneoscleral membrane. It is affected by metabolic, genetic and environmental conditions, as well as myopia and aging [1,3,4]. Several possible factors have been suggested to promote RGC damage and regenerative failure in POAG, namely, mechanical stress (increased IOP causes damage to RGC axons within the lamina cribrosa, thereby interrupting axonal transport), glutamate and zinc toxicity, mitochondrial dysfunction, vascular dysregulation, retinal ischemia and oxidative stress [1,5,6,7]. Recent findings highlighted the pathogenic role of low-grade inflammation driven by some of these factors. Thus, ischemia/reperfusion and oxidative stress promote the expression of a number of inflammatory molecules, including nitric oxide, endothelin-1, vascular endothelial growth factor (VEGF), C-reactive protein, matrix metalloproteinases and various cytokines. Inflammatory responses triggered by these molecules, especially on a background of age-related decrease in the blood-retinal barrier, can promote RGC death [8].

Growing evidence indicates the role of lipid mediators of inflammation in glaucoma, specifically arachidonic acid (AA) and its derivative prostaglandins (PGs). PGs belong to the large family of oxylipins, signaling lipids synthesized from omega-3 and omega-6 polyunsaturated fatty acids (PUFAs) via one or more mono- or dioxygen-dependent reactions catalyzed by cyclooxygenases (COXs), lipoxygenases (LOXs) or cytochrome P450 monooxygenases (CYPs) as well as via the non-enzymatic pathways [9]. PGs regulate many biological processes, including inflammatory responses, pain syndrome and neuronal survival (for review, see [10,11]). Their biosynthesis involves two rate-limiting steps: the production of AA by phospholipase A2 (PLA2) and its conversion into PGH2 by cyclooxygenases, COX-1 and COX-2. PGH2 can be further metabolized into PGD, PGE, PGF2 or other oxylipins [9]. It is found that AA exhibits protective effects with respect to RGCs [12]. Consistently, inhibition of the AA-metabolizing enzyme COX-2 enhances the survival of these cells [13,14]. On the other side, PGs, which can be synthesized from AA by this enzyme, produce a prominent IOP-lowering effect by acting on different prostanoid receptors, thereby causing relaxation of TM [15]. Furthermore, they impact the uveoscleral pathway by increasing the intramuscular spaces in the ciliary body, via remodeling of the extracellular matrix by upregulating biosynthesis of matrix metalloproteinases [16,17]. The most pronounced hypotensive activity is shown in the case of PGF2, the analogs of which are currently approved as the front-line medications for the treatment of glaucoma along with carbonic anhydrase inhibitors, beta-blockers, alpha-agonists and parasympathomimetics [15,18,19].

There are indications of the involvement of several other oxylipins in the pathogenesis of glaucoma. For instance, serum levels of linoleic acid (LA) derivatives hydroxyoctadecadienoic acids (HODEs) and AA derivative hydroxyeicosatetraenoic acids (HETEs) were found to be higher in POAG patients [20]. However, to the best of our knowledge, comprehensive analysis of POAG-related alterations in signaling lipids of AH has never been performed. Meanwhile, AH contacts with all eye tissues affected by glaucoma and its content could reflect biochemical changes associated with the disease [21]. Indeed, significant progress in understanding POAG mechanisms has been achieved upon determining metabolomic alterations in AH [22,23]. Recently, using targeted lipidomic analysis, we have demonstrated that the main types of signaling lipids, such as PUFAs, oxylipins and phospholipid derivatives, are secreted in normal AH and their patterns are significantly altered in various ocular disorders, including those affecting the retina [24,25,26].

The main objective of the current study was to evaluate changes in signaling lipids secreted in AH of POAG patients at different stages of the disease and IOP conditions. The study aimed to find correlations between these changes and the available data on the expression of enzymes responsible for the biosynthesis of the revealed lipids thereby predicting signaling pathways in glaucoma. It should be mentioned that basic oxylipin patterns of AH demonstrated similarities with those of the tear fluid (TF) [24], suggesting that the latter can be used as a source of biomarkers in various ocular disorders. This is especially relevant since, in contrast to AH, TF can be collected by a non-invasive procedure. Consistently, there are a number of works reporting characteristic changes in biochemical properties of the tear film in glaucoma, with or without pharmacological treatment [27,28,29]. Given these indications, the current study also included lipidomic analysis of TF obtained from the same POAG patients. Interestingly, stage/IOP-dependent effects in glaucoma were found to be provided by a small set of specific lipid mediators that exhibited similar alterations in AH and TF.

## 2. Materials and Methods

### 2.1. Materials

Lidocaine (2%) and proxymetacaine (0.5%) eye drops were from Moscow Endocrine Plant (Moscow, Russia) and Alcon-Couvreur (Puurs, Belgium), respectively. The Schirmer test tear strips were from Haag-Streit (Bern, Switzerland). Butylated hydroxytoluene (BHT) was from Sigma-Aldrich (St. Louis, MO, USA). The deuterated oxylipins standards 6-keto PGF1α-d4, TXB2-d4, PGF2α-d4, PGE2-d4, PGD2-d4, LTC4-d5, LTB4-d4, 5(S)-HETE-d8, 12(S)-HETE-d8, 15(S)-HETE-d8, Oleoyl Ethanolamide-d4, EPA-d5, DHA-d5 and AA-d8 were from Cayman Chemical (Ann Arbor, MI, USA). Solid-phase lipid extraction cartridge Oasis^®^ PRIME HLB was obtained from Waters (Eschborn, Germany). Other chemicals were from Sigma-Aldrich, Amresco (Solon, OH, USA), or Serva (Heidelberg, Germany) and were at least reagent grade.

### 2.2. Subjects

AH and TF samples were obtained from 38 patients receiving surgical treatment at Helmholtz National Medical Research Center of Eye Diseases (Moscow, Russia). Of these, 14 were control subjects undergoing cataract surgery by phacoemulsification, and 24 were age-matched patients with a clinically established diagnosis of POAG, which had a non-penetrating deep sclerectomy (Table 1). Diagnosis of POAG was made by the experienced ophthalmologist (SYP) based on a comprehensive examination of the optic nerve state (cupping, thinning of the neuroretinal rim, notch formation and disc hemorrhage), which included visometry, biomicroscopy, ophthalmoscopy, perimetry (Heidelberg Edge Perimeter, SAP-II 30-2 program, Heidelberg Engineering, Heidelberg, Germany), Heidelberg retinal tomography (Heidelberg Retina Tomograph 3, Heidelberg Engineering, Heidelberg, Germany) and optical coherence tomography (Spectralis OCT2, Heidelberg Engineering, Heidelberg, Germany). In Heidelberg retinal tomography, the ratio of the diameter of the excavation of the optic nerve head to its diameter (cup/disc ratio) was evaluated. In optical coherent retinal tomography, the true thickness of the rim was determined as the minimum distance from the Bruch’s membrane to the internal limiting membrane. Prior to the surgery, the majority of patients received hypotensive therapy including prostaglandin analogs, beta-blockers, carbonic anhydrase inhibitors and/or alpha-agonists for at least 1 year. During this time, all patients were subjected to IOP monitoring once every 2 months (measurements were taken at the same time in the morning, 9–10 AM) using ICare PRO tonometer (ICare, Vantaa, Finland). Immediately prior to the operation, the patients were assessed as described above, assigning to POAG stages II (moderate POAG) or III (advanced POAG), and grade A, B or C depending on average IOP parameters in accordance with National Glaucoma Guidelines [30] (for detailed characteristics of the experimental groups see also Results section).

The studies were conducted in accordance with the Declaration of Helsinki and the ARVO statement on human subjects and were approved by the local ethical committee of Helmholtz National Medical Research Center of Eye Diseases. All participants signed written informed consent.

### 2.3. AH and TF Collection

AH was taken by the surgeon (SYP) during phacoemulsification or non-penetrating deep sclerectomy under peribulbar (2% lidocaine) and topical (proxymetacaine 0.5% eye drops) anesthesia as described previously [24]. Briefly, a paracentesis incision through the cornea was made using a 1.2 mm long single-use knife and 50 μL of AH was aspirated using a syringe, mixed with 0.05% BHT (50:1 *v/v*), aliquoted and stored at −80 °C.

TF was collected in patients on the day of the surgery using gauged Schirmer’s test paper strips without anesthesia or tear stimulation. In all subjects, the procedure was performed under identical conditions, namely thirty minutes after awakening, the fasted state, same medical personnel, same air and light conditions. The strip was allowed to become moistened for 10 mm under the lower eyelid, and the wet fragment was cut off, placed to 1 mL of 95% *v/v* water-methanol solution containing 0.1% *v/v* BHT and stored at −80 °C.

### 2.4. Lipid Extraction

Lipidomic analysis was performed as described in our recent study [24]. Briefly, TF samples were released from Schirmer’s test paper fragments, whereas AH samples were diluted by 1 mL of 95% *v/v* water-methanol solution. After that, both solutions were mixed with deuterated internal standard solutions, centrifuged (12,000× *g*, 3 min), the supernatant was mixed with 0.1% acetic acid and loaded onto solid-phase lipid extraction cartridge (Oasis ^®^ PRIME HLB cartridge (60 mg, 3 cc)). Solid-phase extraction was performed using cartridge manifold VacElut (Agilent, Santa Clara, CA, USA) with a laboport mini-pump (KNF, Hamburg, Germany). The cartridge was washed with 15% methanol containing 0.1% formic acid and lipids were sequentially eluted with 500 μL of anhydrous methanol and 500 μL of acetonitrile. After the extraction, the samples were concentrated by evaporation of the solvent under a gentle stream of nitrogen, reconstituted in 50 μL of 90% methanol and stored at −80 °C until the further analysis.

### 2.5. UPLC-MS/MS Analysis

For the identification of lipid mediators in the TF and AH samples, the respective lipid extracts were analyzed using 8040 series UPLC-MS/MS mass spectrometer (Shimadzu, Kyoto, Japan) provided by RUDN University Strategic Academic Leadership Program. Multiple-reaction monitoring mode at a unit mass resolution was used for both the precursor and product ions. The lipid compounds were separated by reverse-phase UPLC (injection volume 20 μL) using Phenomenex C8 column (Kinetex® 2.6 µm C8 100 Å, LC Column 150 mm × 2.1 mm) with the flow rate of 0.4 mL/min and temperature of the sample cooler and the column of 5 °C and 40 °C, respectively. The elution was performed using acetonitrile gradient in 0.1% (*v/v*) formic acid. The separated lipids were subjected to MS analysis employing an electrospray ionization source operating in both positive and negative ion modes using nitrogen as the nebulizer gas. The selected molecular ions were fragmentized in the gas phase by collision-induced dissociation and analyzed by MS/MS. The parameters of mass spectrometry were set as follows: nebulizer gas flow, 3 L/min; drying gas, 10 L/min; heat block temperature, 400 °C; desolvation line temperature, 250 °C; collision induced dissociation pressure, 230 kPa. The target lipids were identified and quantified by comparing their UPLC, MS and MS/MS parameters with the respective data obtained for deuterated internal standard compounds employing Lipid Mediator Version 2 software (Shimadzu, Kyoto, Japan). The concentrations of the lipids in each AH and TF sample are presented in Supplementary Table S1.

### 2.6. Statistics

Comparison of demographic and health characteristics between the groups was performed using the two-sample two-sided t-test in case of numerical parameters (age, IOP and cup/disc ratio) or two-sided z-test in case of ratio parameters (gender, refraction anomalies and comorbidities).

Comparison of signaling lipids contents between the groups was made using two alternative methods, namely analysis of covariance (ANCOVA) and partial least square discriminant analysis (PLS-DA). ANCOVA was performed using rstatix package. Pairwise comparisons of mean concentrations of single lipids were performed using emmeans_test function, considering age and gender as covariates. This analysis was followed by Bonferroni–Holm correction for multiple comparisons; *p* < 0.05 was considered as a cut-off threshold in the volcano plot built using EnchancedVolcano package.

PLS-DA was conducted using the mixOmics package. The concentrations of single lipids were mean-centered, and unit variance was scaled. Leave-one-out cross-validation was used to estimate an optimal number of components in each model. The overall error, balanced error rate and AUC were taken into consideration for model evaluation. After the building of PLS-DA models with optimal components, each lipid was assigned a VIP score denoting a weighted sum of squares of the PLS loadings regarding the explained variation in each projection. A cutoff for VIP scores was accepted as 1.5 according to the Metabolomics Standard Initiative (MSI; level 1) [31].

The impact of comorbidities and treatment regimens on signaling lipids contents was evaluated using two-way ANOVA, where one categorical variable was the control or glaucoma group, whereas another variable was the presence or absence of the particular treatment or comorbidity.

### 2.7. Transcriptome Analysis

Raw data were obtained from Sequence Read Archive (SRA) using SRA toolkit. The transcripts were annotated using reference human transcriptome GRCh38.v32. Indexing and quantification of the reads were performed by Salmon tool with useVBOpt, seqBias, and validateMappings flags [32]. The import and summarization of the transcripts to the gene-level were conducted by tximport R package [33]. Data were normalized using the median of ratio approach and analyzed for outliers by principal component analysis (PCA) plots with hierarchical samples classification. Differential expression analysis was performed using DESeq2 package [34]. Differentially expressed genes (DEGs) were determined using standard criteria, namely *p*-value (adjusted with Benjamini–Hochberg procedure) < 0.05 and fold change difference more than twice. Visualization of the results was performed using EnchancedVolcano package.

## 3. Results

### 3.1. Characteristics of the Experimental Groups

The study involved a total of 24 patients with POAG and 14 cataract patients without a history of glaucoma that were used as controls. The detailed demographic characteristics and comorbidities of the participants are summarized in Table 1 and Supplementary Table S1. The total POAG group predictably demonstrated a higher mean cup-to-disc ratio and IOP value than the control group, in the absence of differences in gender proportion and age. Glaucoma patients did not exhibit statistically significant differences in the prevalence of arterial hypertension, coronary heart disease, diabetes mellitus or myopia. To account for the severity of glaucoma and IOP levels, the members of the total POAG group were further divided into four stage-dependent POAG subgroups by assigning them to Stages II-III of the disease (patients with Stage I (mild POAG) and Stage IV (severe POAG) were absent in the cohort) and grades A (IOP ≤ 21 mmHg), B (IOP of 22–28 mmHg) and C (IOP ≥ 29 mmHg) [30]. Stage II (moderate glaucoma) was assigned to patients with changes in the paracentral section of the visual field (narrowed by more than 10° in the nasal hemifield) and regional optic disc cupping. The average cup-to-disc ratio in these patients was approximately 0.7 (Table 1). Stage III (advanced glaucoma) was assigned to patients with concentrically narrowed visual field (less than 15° from the fixation in at least one segment) and subtotal optic disc cupping. In these individuals, the average cup-to-disc ratio tended to 0.85 (Table 1). The number and age of patients in the stage-dependent POAG subgroups were generally similar, thereby enabling a reasonable comparison.

### 3.2. Profile of Signaling Lipids Secreted in AH

The content of signaling lipids in AH of both control individuals and POAG patients was assessed by means of an MS-based targeted lipidomic technique focusing on PUFAs, oxylipins and phospholipid derivatives. A total of 22 compounds were identified (Table 2). The pool of PUFAs consisted of eicosapentaenoic (EPA), docosahexaenoic (DHA) acids (omega-3 PUFA) and AA (omega-6 PUFA). The AA-derivative oxylipins included thromboxane B3 (TXB3), leukotriene B4 (LTB4), 20-carboxy-LTB4, 19-HETE and 20-HETE, as well as an inseparable mixture of prostaglandins PGA2 and PGJ2. The second large set of oxylipins was represented by LA derivatives, namely hydroxyoctadecadienoic (9-HODE/13-HODE), oxooctadecadienoic (9-KODE/13-KODE), epoxyoctadecamonoenoic (9,10-EpOME/12,13-EpOME) and dihydroxyoctadecamonoenoic (9,10-DiHOME/12,13-DiHOME) acids. In addition, we detected α-linolenic acid (ALA)-derivative hydroxyoctadecatrienoic acids (9-HOTrE and 13-HOTrE) and two phospholipid derivatives, oleoylethanolamide (OEA) and lyso-platelet-activating factor (lyso-PAF).

### 3.3. POAG-Related Alterations in Signaling Lipids of AH

**Figure 1:**
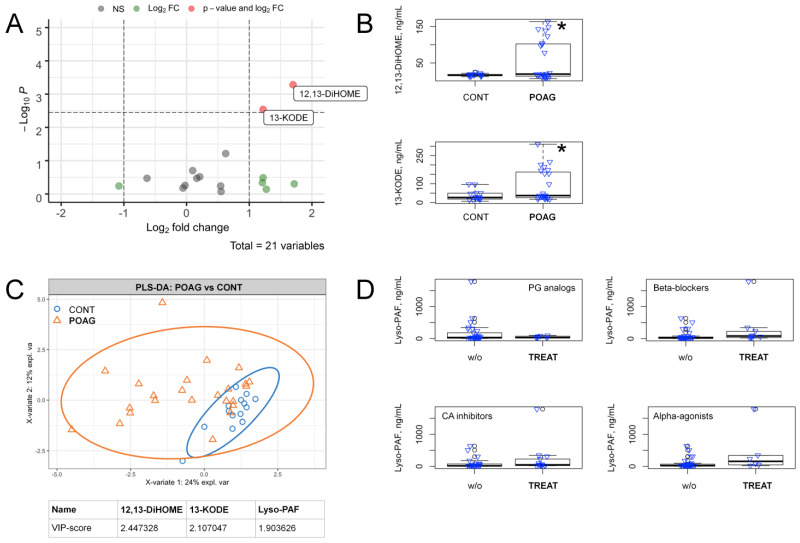
Alterations in the content of signaling lipids in AH in the total cohort of POAG patients. (**A**) volcano plot presents compounds discriminating the total POAG and control groups. The X-axis indicates a log2 fold change of age and gender-adjusted concentrations of the identified compounds in POAG patients as compared to the control individuals. The Y-axis indicates -log10 *p*-values with the cut-off calculated according to Bonferroni correction. Compounds that changed less than twofold (Log2 FC) or demonstrated non-significant (NS) alterations (*p* > 0.05) are indicated in green and gray colors, respectively. The significantly changed compounds (*p* < 0.05) are denoted in red color (value and Log2 FC). (**B**) the box plots demonstrating concentrations of metabolites exhibiting significant changes in glaucoma patients (POAG) as compared with control individuals (CONT). * *p* < 0.05 (adjusted for multiple testing). (**C**) the results of partial least square discriminant analysis (PLS-DA) revealing compounds distinguishing total glaucoma patients (POAG) from the control individuals (CONT). The explained variance of each component is indicated on the axes. Variable importance in projection (VIP) scores exceeding a cutoff value of 1.5 are considered. (**D**) the box plots illustrating pairwise comparisons of lyso-PAF concentrations in AH of POAG patients with (TREAT) or without (w/o) anti-glaucoma treatment using prostaglandins (PG) analogs, beta-blockers, carbonic anhydrase (CA) inhibitors or alpha-adrenergic agonists. In all cases *p* > 0.05.

To identify AH lipids discriminating the total POAG and control groups, we first performed pairwise comparisons for the concentration of each of the revealed compounds in an age and gender-adjusted manner, using ANCOVA. As can be seen from the resulting volcano plot with Holm–Bonferroni correction, the difference between the groups was provided by only two LA-derivative oxylipins, 12,13-DiHOME and 13-KODE (Figure 1A), which demonstrated a prominent increase in glaucoma (Figure 1B). Since the t-test only considers each variable separately, the data were additionally recalculated using partial least squares-discriminant analysis (PLS-DA), which employs a multidimensional space, thereby more efficiently differentiating the multicollinear variables [35]. To reveal significant differences, a cutoff value of 1.5 for the calculated variable importance in projection (VIP) scores was used [31]. Remarkably, the evaluations using PLS-DA revealed the same oxylipins 12,13-DiHOME and 13-KODE, complemented by phospholipid derivative lyso-PAF, which was also upregulated in POAG (Figure 1C).

Since the members of the total POAG group received complex hypotensive therapy, we next verified if the revealed lipidomic changes stemmed from the effects of the respective antiglaucoma agents, namely prostaglandins analogs, beta-blockers, carbonic anhydrase inhibitors or alpha-adrenergic agonists. The pairwise comparisons of the data regarding AH concentration of lyso-PAF for POAG patients, receiving or not receiving each kind of the abovementioned therapies, revealed no significant differences (Figure 1D). The same results were obtained for 12,13-DiHOME and 13-KODE (as well as for the other POAG-dependent lipids revealed in the study, see below; Supplementary Figure S1). In addition, no significant influence was found from systemic diseases, such as arterial hypertension, coronary heart disease and diabetes mellitus (Supplementary Table S2). Thus, the changes in these compounds represented a POAG-specific effect.

### 3.4. POAG Stage and IOP-Dependent Alterations in Signaling Lipids of AH

**Figure 2:**
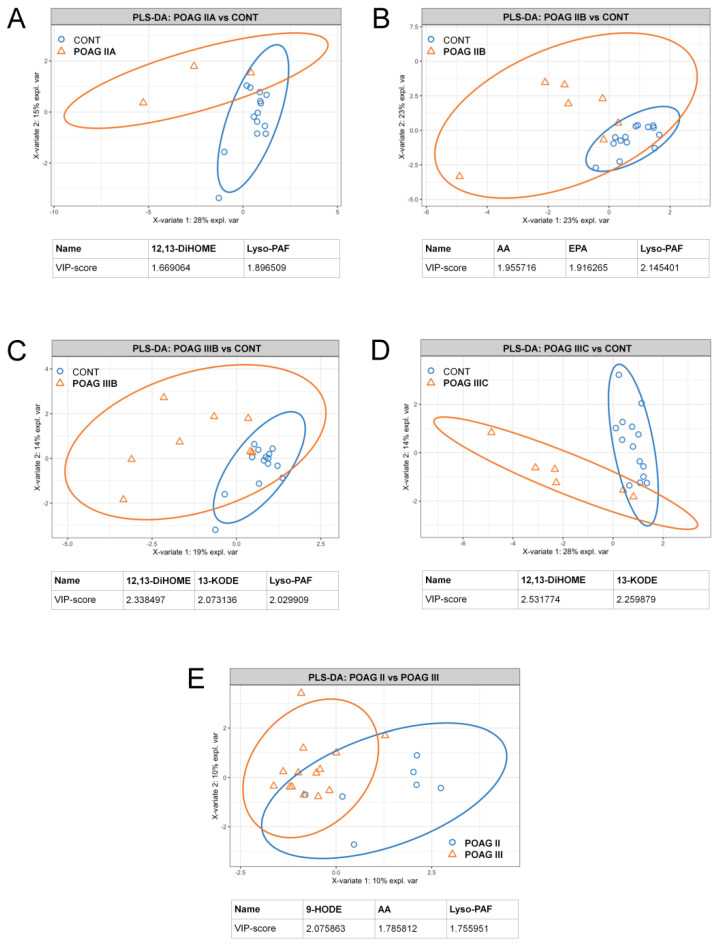
Stage/IOP-dependent alterations in the content of signaling lipids in AH of POAG patients. The results of partial least square discriminant analysis (PLS-DA) revealing AH compounds distinguishing glaucoma patients with Stage IIA (**A**), Stage IIB (**B**), Stage IIIB (**C**) or Stage IIIC (**D**) POAG from the control individuals (CONT). The PLS-DA data regarding comparison of AH contents of signaling lipids in patients with different POAG stages regardless IOP levels are also presented (**E**). The explained variance of each component is indicated on the axes. Variable importance in projection (VIP) scores exceeding a cutoff value of 1.5 are considered.

Given that POAG is a slowly progressive disorder, we next identified signaling lipids of AH exhibiting dynamic changes following its development. We employed PLS-DA to compare the data for each of the stage/IOP-dependent POAG subgroups (see Table 1) with those for the control group (Figure 2A–D). In addition, we compared all Stage II patients with all Stage III patients in this respect (Figure 2E). (The data regarding the net effect of IOP-dependent changes in the signaling lipidome of AH and TF are also provided; see Supplementary Figure S2). In general, the analyzed sets demonstrated an increasing degree of discrepancy with the growth of glaucoma severity. The alterations were most often provided by the same compounds, namely 12,13-DiHOME, 13-KODE and lyso-PAF, although in some cases, we additionally registered differences in AA and 9-HODE contents.

Notably, although most of these compounds were accumulating in AH with POAG development, 9-HODE demonstrated the opposite trend. This effect may be related to oxidation and thereby depletion of HODEs (precursor), yielding KODEs (product) as the latter being increased in glaucomatous AH. Indeed, similarly to 13-KODE, 9-KODE was increased in some patients with Stage III POAG (Supplementary Table S1 and Figure S3). 

Overall, we concluded that linoleic acid derivatives 12,13-DiHOME and 9- and 13-KODE, as well as AA and lyso-PAF, are the main signaling lipids increased in AH following POAG development.

### 3.5. Alterations in Oxylipin Content of TF, Associated with POAG

**Figure 3:**
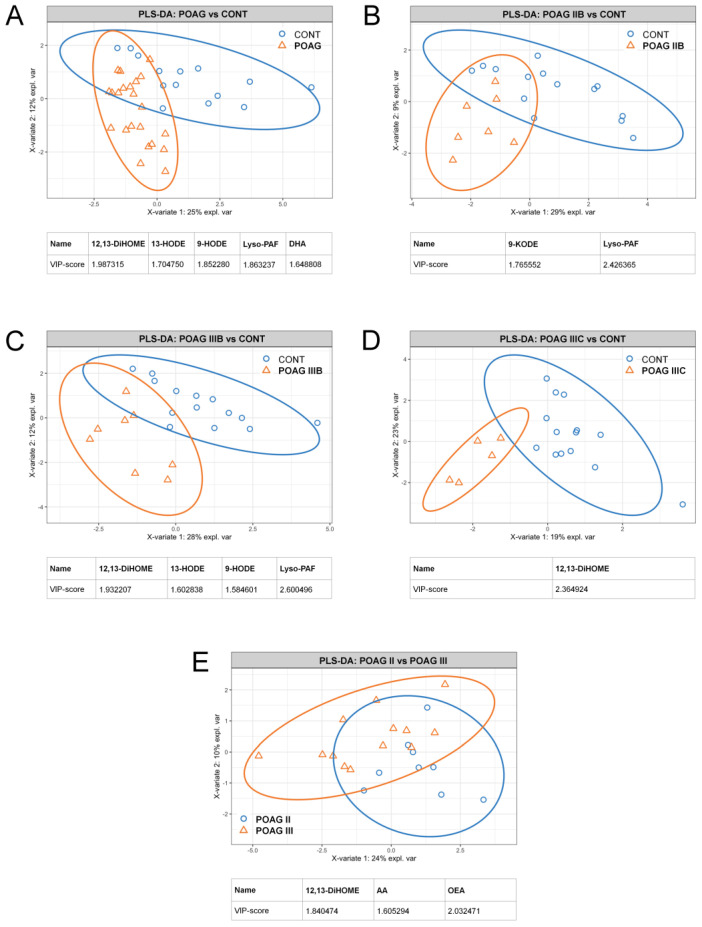
Total and stage/IOP-dependent alterations in the content of signaling lipids in TF of POAG patients. The results of partial least square discriminant analysis (PLS-DA) revealing TF compounds distinguishing total glaucoma patients (**A**) and patients with Stage IIB (**B**), Stage IIIB (**C**) or Stage IIIC (**D**) POAG from the control individuals (CONT). The PLS-DA data regarding comparison of TF contents of signaling lipids patients with different POAG stages regardless IOP levels are also presented (**E**). The explained variance of each component is indicated on the axes. Variable importance in projection (VIP) scores exceeding a cutoff value of 1.5 are considered.

Since TF is often regarded as a source for biomarkers of various ocular disorders, we employed the approaches described above to analyze POAG-associated changes in TF collected from the same patients and the control group. The targeted lipidomic analysis of TF allowed for the identification and quantification of 22 lipid compounds, representing the same set as in the case of AH (see Table 2). Furthermore, the difference between all POAG groups and control in TF lipidome was provided mainly by the same signaling lipids, namely 12,13-DiHOME, 9- and 13-HODE/KODE, AA and lyso-PAF (Figure 3). Similarly to AH, glaucomatous TF exhibited a decrease in HODEs (precursor) accompanied by an increase in KODEs (product). The general exception was DHA, which demonstrated glaucoma-specific decrease only in TF, particularly in the pairs ‘total POAG versus control’ (Figure 3A) and ‘Stage IIA versus control’ (data not shown). As in the case of AH, the treatment with antiglaucoma agents had no effect on patterns of signaling lipids in TF (Supplementary Figure S4).

### 3.6. Bioinformatics Analyses

**Figure 4:**
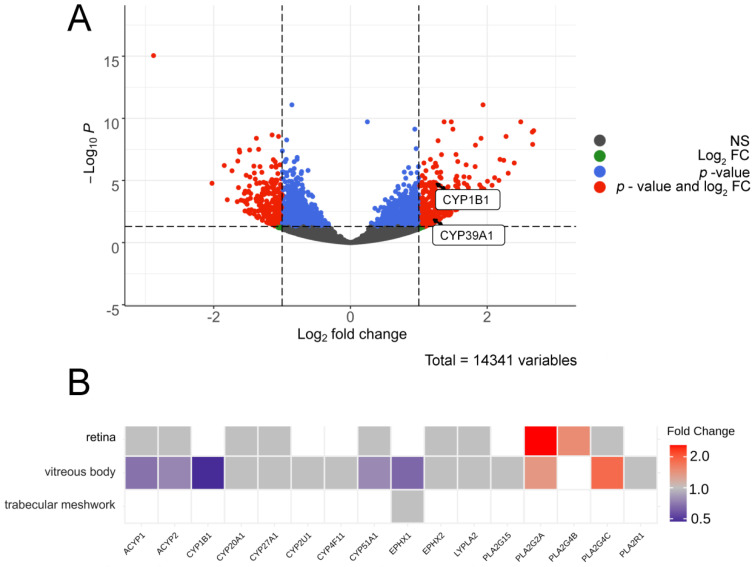
Bioinformatic analysis of enzymatic pathways responsible for production of the revealed POAG-specific lipid mediators in AH. (**A**) volcano plot indicating DEGs corresponding to signaling lipids-synthesizing enzymes in TM cells underwent long-term perfusion at high pressure according to analysis of the transcriptomic data 37. The X-axis indicates a log2 fold change in levels of transcripts in TM cells perfused at high pressure for 3 h as compared to 5 h. The Y-axis indicates -log10 *p*-values with the cut-off calculated according to Bonferroni correction. mRNAs that changed less than twofold (Log2 FC) or demonstrated non-significant (NS) alterations (*p* > 0.05) are indicated in green and gray colors, respectively. The significantly changed transcripts (*p* < 0.05) are denoted in red color (value and Log2 FC). (**B**) a clustered image map illustrating changes in expression of signaling lipids-synthesizing enzymes in the retina, vitreous body and trabecular meshwork of POAG patients determined from analysis of proteomic data. The map is built using the Euclidean distance and complete linkage clustering algorithm. Each entry is colored according to its fold change, rows represent tissues, and columns represent enzymes. Abbreviations: ACYP: acylphosphatase-2; CYP1B1: cytochrome P450 1B1, CYP20A1: cytochrome P450 20A1; CYP27A1: cytochrome P450 27A1; CYP2U1: cytochrome P450 2U1; CYP4F11: cytochrome P450 4F11; CYP51A1: cytochrome P450 51A1; EPHX1: epoxide hydrolase 1; EPHX2: epoxide hydrolase 2; LYPLA2: acyl-protein thioesterase 2; PLA2G15: phospholipase A2 G15; PLA2G4B: phospholipase A2 G4B; PLA2G4C: phospholipase A2 G4C; PLA2R1: phospholipase A2 R1.

To predict enzymatic pathways responsible for the production of the revealed POAG-specific lipid mediators in AH, we next analyzed datasets describing the glaucoma-related alterations in transcriptomes and proteomes of the affected ocular tissues. In particular, we addressed changes in TM, retina and vitreous body as these tissues can secret signaling lipids into AH, while being the most affected by glaucoma [1,36]. In the absence of proteomic findings regarding POAG-related alterations in human TM tissue, we first analyzed RNA-Seq data obtained in the case of primary human TM cells cultured under conditions of increasing substrate stiffness, a cellular model simulating one of the main pathogenetic processes in glaucoma patients [37]. The focus was made on the enzyme families involved in the biosynthesis of the reveled signaling lipids, namely CYPs/epoxide hydrolases (DiHOMEs), LOXs (HODE/KODE) and PLA2s (AA and lyso-PAF). The performed RNA-Seq differential expression analysis revealed over 590 DEGs, only two of which, namely CYP1B1 and CYP39A1, met the above-mentioned criteria and exhibited significant growth with an increase in the duration of the perfusion (Figure 4A).

To complete analysis of the ocular tissues affected by glaucoma, we next performed the proteomic data analysis regarding the retina and vitreous body samples collected from POAG patients [38,39]. In addition, we assessed the proteome of TM obtained from human anterior segments perfused at high pressure using an ex vivo organ culture system [40]. The results are presented as a heat map illustrating POAG-associated changes in tissue content of the analyzed enzymes (Figure 4B). Among the 16 proteins belonging to the examined families (see above), only two PLA2s, PLA2G2A and PLA2G4B, were found to be increased in the glaucomatous retina. Interestingly, PLA2G2A and PLA2G4C were also elevated in the vitreous body. However, it additionally exhibited decreased content of epoxide hydrolase-1 (EH-1), CYP1B1 and CYP51A1. It should be added that none of the 16 targeted proteins were upregulated in TM in the ex vivo model, and no changes in expression levels of LOXs were found in all analyzed tissues (data not shown).

## 4. Discussion

This study is the first to purposefully address alterations in signaling lipidome of AH in POAG patients. Previous works on the subject were focused mainly on the general contents of phospholipids and sphingolipids, whereas the patterns signaling lipids (i.e., oxylipins) in glaucomatous AH remained obscure [41,42]. Here, using the MS-based methodology developed for targeted lipidomic analysis of the eye fluids in our recent works [24,25,26,43], we identified a total of 22 lipid mediators, among which there were three PUFAs, 17 oxylipins and two phospholipid derivatives. The nomenclature of these compounds generally coincided with those we had previously detected in AH and TF of individuals without glaucoma and intraocular inflammation [24]. Interestingly, we have demonstrated that there were pronounced specific alterations in signaling lipids of AH, which can be attributed to POAG pathogenesis. The stage/IOP-dependent effects in glaucoma were provided by a small set of mediators, namely linoleic acid derivatives 12,13-DiHOME, 9-HODE/KODE and 13-HODE/KODE, as well as AA and lyso-PAF.

LA-derived oxylipin 12,13-DiHOME can be considered as the most relevant biomarker of POAG since it was most frequently encountered in our analysis as a compound providing the difference between the control and glaucoma groups for both AH and TF. It belongs to a recently identified class of lipids called lipokines that act as signaling molecules modulating systemic metabolism. 12,13-DiHOME becomes upregulated in response to cold exposure or physical exercise and its effect is associated with improved metabolism such as increased fatty acid oxidation and uptake [44,45]. The common biosynthetic pathway for 12,13-DiHOME involves two steps, LA oxidation by CYPs to form the epoxy derivative 12,13-EpOME and its subsequent dihydroxylation catalyzed by EH-1 or EH-2 [9,44,46]. Even though 12,13-DiHOME has never been associated with glaucoma, the existing transcriptomic and proteomic data regarding POAG-related alterations in ocular tissues generally support increased production and secretion of this oxylipin in AH and TF. Thus, our bioinformatic analysis revealed upregulation of two CYP enzymes, CYP1B1 and CYP39A1, in TM cells model of glaucoma pathogenesis [37]. Consistently, another relative enzyme, CYP2S1, increased in rat retina in the glaucoma model based on optic nerve transaction [47]. At the same time, CYP1B1 was found to be decreased in vitreous body of POAG patients (Figure 4B) [38]. Remarkably, CYP1B1 is known as the most frequent causative gene in primary congenital glaucoma and its mutations were often found in POAG patients suggesting the existence of a common CYP1B1-mediated mechanism of both variants of the disease [48]. Although there are no direct indications on the involvement of CYP1B1 in 12,13-DiHOME synthesis, the latter may represent signaling lipid mediating pathological effects associated with aberrations in CYP1B1.

Notably, CYPs are known as oxidative stress markers as they are involved in the processing of different toxic metabolites with formation of reactive oxygen species, such as superoxide anion, hydrogen peroxide and hydroxyl radical [49]. Consistently, the upregulation of CYPs, evidenced by a stable increase in their product 12,13-DiHOME, may be an indication of oxidative stress in the ocular tissues affected by POAG. Although producing beneficial metabolic effects at low doses, increased levels of 12,13-DiHOME can promote uncoupling of the mitochondrial electron transport chain and oxidative stress [50], which may lead to RGC death. The development of oxidative stress in POAG is further evidenced by growth of the other signaling lipids of AH, 9-HODE/KODE and 13-HODE/KODE, observed in our study. These metabolites can be biosynthesized from LA via two alternative mechanisms, namely LOXs-dependent pathways or free radical-induced nonenzymatic oxidation [9]. Given that no POAG-associated alterations in LOXs were found in the affected ocular tissues (TM, retina or vitreous body) [37,38,39], one can expect oxidative stress as a major driving force for production of HODEs/KODEs found in glaucomatous AH. Indeed, these oxylipins are widely regarded as biomarkers of oxidative stress in different tissues [51,52]. Furthermore, the levels of 9- and 13-HODEs are higher in blood of patients with POAG and exfoliation syndrome [20,53]. These observations agree with our data, as HODEs are precursors of KODEs and we observed an increase in KODEs in glaucomatous AH, which is at least partially produced by plasma ultrafiltration [21]. It should be also noted that CYP1B1 and its products may play a specific role in redox-related mechanisms of glaucoma: this enzyme participates in regulation of redox homeostasis and its absence may reduce the tolerance of TM and retina to oxidative stress, thereby causing developmental defects and congenital glaucoma, or POAG [54]. Taken together, these data confirm the critical role of oxidative stress in glaucoma pathogenesis [55].

Another important observation is the prominent POAG-associated increase in AH content of AA and lyso-PAF. These compounds represent co-products of hydrolysis of phospholipids (such as 1-O-alkyl-2-acyl-GPC) catalyzed by PLA2s [56]. Consistently, analysis of proteomic data recognized such phospholipases (PLA2G2A, PLA2G4B and PLA2G4C) as the major type of signaling lipid metabolism enzymes increased in the retina and vitreous body of the POAG patients (Figure 4B) [38,39]. Moreover, PLA2G2A was shown to be significantly upregulated in the other ocular tissues, such as TM and conjunctiva of such patients [57,58]. Being formed within the same pathway, AA and lyso-PAF can produce different physiological effects. LysoPAF is acetylated by lysoPAF acetyltransferases yielding physiologically active PAF, which triggers platelet aggregation and inflammatory responses through activation of the respective receptors [56]. PAF seems to play crucial role in ocular inflammation and glaucoma [59,60]. Thus, it is known to promote ocular hypertension and may contribute to RGC damage via enhancing glutamate release and excitotoxicity in the retina [61,62]. By contrast, AA exhibits protective effects in respect to RGC and could be processed by COXs forming PGs, which increase AH outflow through both TM and uveoscleral pathways [12,15,16,17]. Notably, we detected neither prostaglandins nor COXs elevations in POAG, suggesting that PLA2/AA pathway is settled on AA production.

Overall, the augmentation of the content of IOP-increasing and cytotoxic mediators PAF and 12,13-DiHOME, together with oxidative stress-related oxylipins HODEs/KODEs, represent a part of the pathophysiological picture of POAG manifesting in the signaling lipidome of AH. An increase in RGC-defensive mediator AA may correspond to a compensatory neuroprotective mechanism, although the apparent arrest of its processing into IOP-decreasing PGs may inversely contribute to POAG pathogenesis. In any case, the identified lipid mediators can be considered as specific biomarkers of glaucoma, especially since their similar patterns were detected in TF, which can be collected from patients by a non-invasive procedure. Furthermore, the targeted modulation of signaling pathways involving these lipids can provide novel insights into glaucoma treatment. Prostaglandin analogs widely employed in POAG therapy are known to inhibit PAF activity [63], suggesting that their IOP-decreasing activity may involve PAF inhibition. The revealed increase in 12,13-DiHOME may be suppressed using novel EH inhibitors, which are currently being trialed as a pharmacotherapy of different ocular diseases [64]. Finally, our findings confirm a role of oxidative stress in POAG and provide an additional rationale for the inclusion of antioxidants in complex treatment of the disease [55]. Nevertheless, the exact mechanisms mediated by the identified signaling lipids in glaucoma, as well as approaches to manage these mechanisms, are yet to be determined.

## Figures and Tables

**Table 1:** Characteristics of control and POAG groups.

Parameter		Control Group	Stage-Dependent POAG Subgroups (IOP)				Total POAG Group
			IIA (≤21 mmHg)	IIB (22–28 mHg)	IIIB (22–28 mHg)	IIIC (≥29 mmHg)	
Number of partici		14	3	7	8	6	24
Mean age ± SD ^1^, years		65.64 ± 13.77	67.0 ±2.65	68.86 ± 4.18	71.62 ± 6.8	63.33 ± 6.65	68.17 ± 9.35
Gender, %	Male	21.43	0.0	14.29	50.0	33.33	29.17
	Female	78.57	100.0	85.71	50.0	66.67	70.83
IOP ± SD, mmHg		14.9 ± 1.8	17.7 ± 1.7	24.7 ± 2.1	25.5 ± 1.8	33.5 ± 3.4	26.3 ± 1.9
Cup/disc ratio		0.54 ± 0.12	0.73 ± 0.05	0.71 ± 0.04	0.84 ± 0.09	0.85 ± 0.06	0.79 ± 0.06
Refr., %	Myopia	42.86	33.33	42.86	12.5	16.67	25.00
Comorbidities, %	HTN ^2^	42.86	66.67	42.86	50.0	50.0	50.00
	CHD ^3^	14.29	33.33	28.57	75.0	0	37.50
	DM ^4^	7.14	0	28.57	0	16.67	12.50
Treatment, %	PG analogs	0	0	0	62.5	50.0	33.33
	β-blockers	0	100	57.14	37.5	33.33	37.50
	CAI ^5^	0	100	71.43	50.0	66.67	54.16
	α-agonists	0	0	42.86	12.5	33.33	25.00

^1^ SD, standard deviation; ^2^ HTN, arterial hypertension; ^3^ CHD, coronary heart disease; ^4^ DM, diabetes mellitus; ^5^ CAI, carbonic anhydrase inhibitor.

**Table 2:** Nomenclature of the lipid compounds detected in AH.

#	Name	Short Name	Precursor	Pathway
**PUFAs**				
1	Arachidonic acid	AA	Phospholipids	PLA2
2	Docosahexaenoic acid	DHA	Diet, EPA	-
3	Eicosapentaenoic acid	EPA	Diet, ALA	-
**Oxylipins**				
4	Leukotriene B4	LTB4	AA	LOX
5	20-carboxy-leukotriene B4	20-carboxy-LTB4	AA	LOX
6	19-hydroxyeicosatetraenoic acid	19-HETE	AA	LOX
7	20-hydroxyeicosatetraenoic acid	20-HETE	AA	LOX
8	Prostaglandin A2	PGA2	AA	COX
9	Prostaglandin J2	PGJ2	AA	COX
10	Thromboxane B3	TXB3	AA	COX
11	9-hydroxyoctadecadienoic acid	9-HODE	LA	LOX
12	9-oxo-octadecadienoic acid	9-KODE	LA	LOX
13	13-hydroxyoctadecadienoic acid	13-HODE	LA	LOX
14	13-oxo-octadecadienoic acid	13-KODE	LA	LOX
15	9,10-dihydroxyoctadecamonoenoic acid	9,10-DiHOME	LA	CYP
16	9,10-epoxyoctadecamonoenoic acid	9,10-EpOME	LA	CYP
17	12,13-dihydroxyoctadecamonoenoic acid	12,13-DiHOME	LA	CYP
18	12,13-epoxyoctadecamonoenoic acid	12,13-EpOME	LA	CYP
19	9-hydroxyoctadecatrienoic acid	9-HOTrE	ALA	LOX
20	13-hydroxyoctadecatrienoic acid	13-HOTrE	ALA	LOX
**Phospholipid Derivatives**				
21	Lyso-platelet-activating factor	Lyso-PAF	Phospholipids	PLA2
22	Oleoylethanolamine	OEA	Phospholipids	NAPE-PLD

## Supplementary Materials

The following are available online at https://www.mdpi.com/article/10.3390/biology10070658/s1, Figure S1: Effect of antiglaucoma treatments on AH content of signaling lipids in total POAG group. Figure S2: The PLS-DA data regarding comparison of AH (A) and TF (B) contents of signaling lipids in patients with different IOP levels regardless POAG stage. Figure S3: Stage-dependent alterations in the content of 9-KODE in AH of POAG patients. Figure S4: Effect of antiglaucoma treatments on TF content of signaling lipids in total POAG group. Table S1: Demographic characteristics, medical records and raw lipidomic data of all individuals participated in the study. Table S2: *p*-values for pairwise comparisons (using ANCOVA with Bonferroni correction) of AH and TF concentrations of each POAG-dependent lipid in patients with or without different systemic disease.
