# Supplementary figures and images for: Targeted Lipidomic Analysis of Aqueous Humor Reveals Signaling Lipid-Mediated Pathways in Primary Open-Angle Glaucoma
Source: Biology (Basel). 2021 Jul 13;10(7):658. doi: 10.3390/biology10070658 (PMC8301454; doi:10.3390/biology10070658)

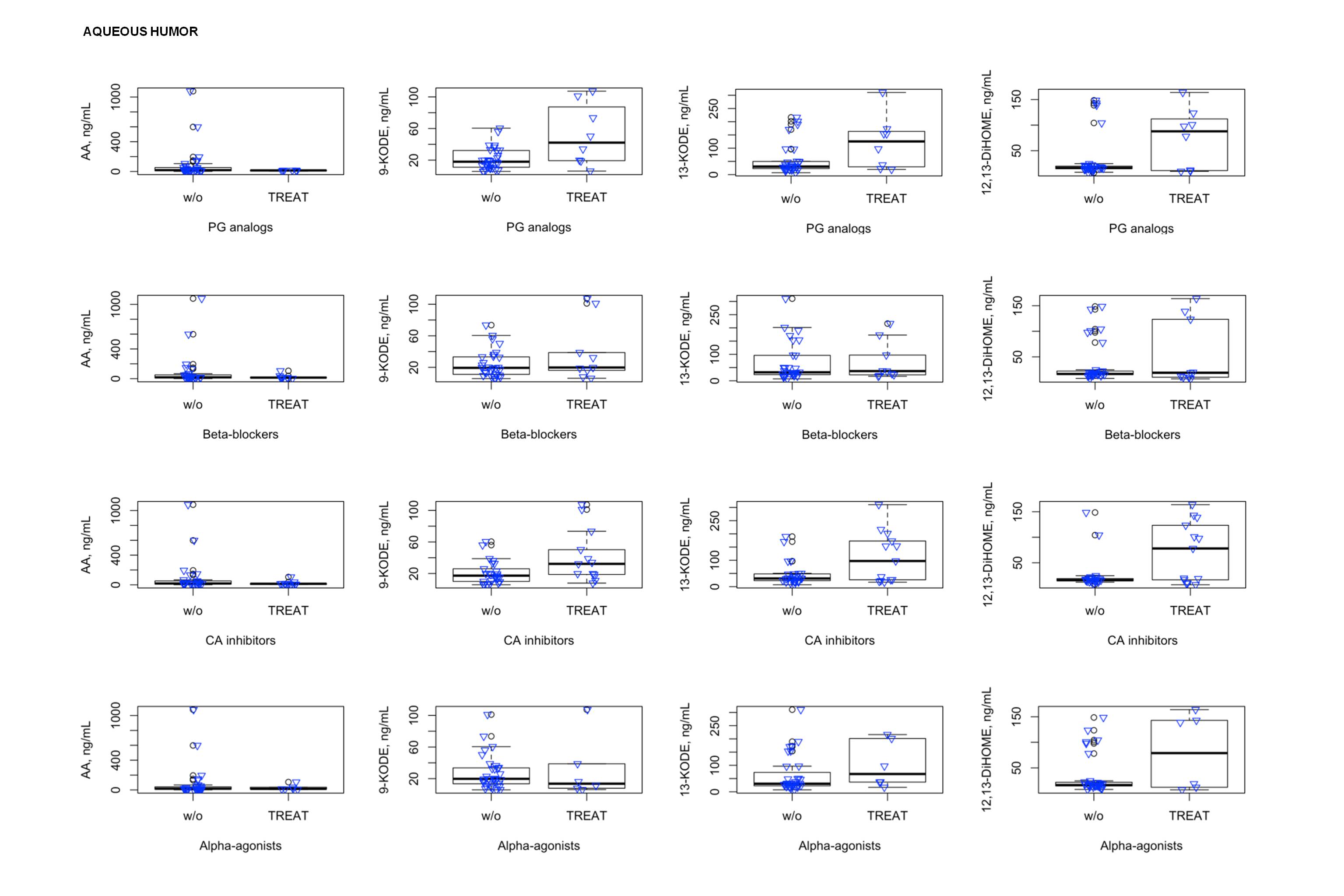

Supplement: Supplementary file 1 [file biology-10-00658-s001.zip › biology-1268784-supplementary/Azbukina et al. 2021 Figure S1.jpg]

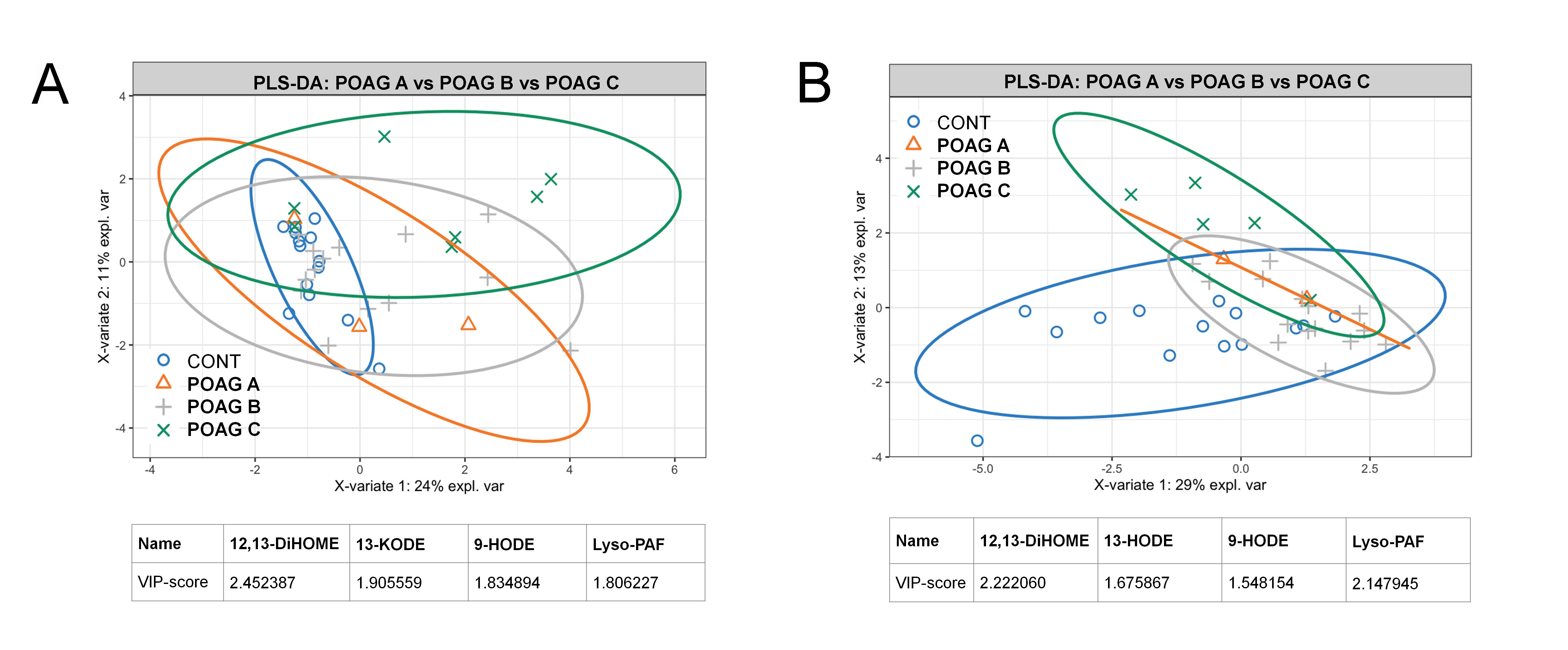

Supplement: Supplementary file 1 [file biology-10-00658-s001.zip › biology-1268784-supplementary/Azbukina et al. 2021 Figure S2.jpg]

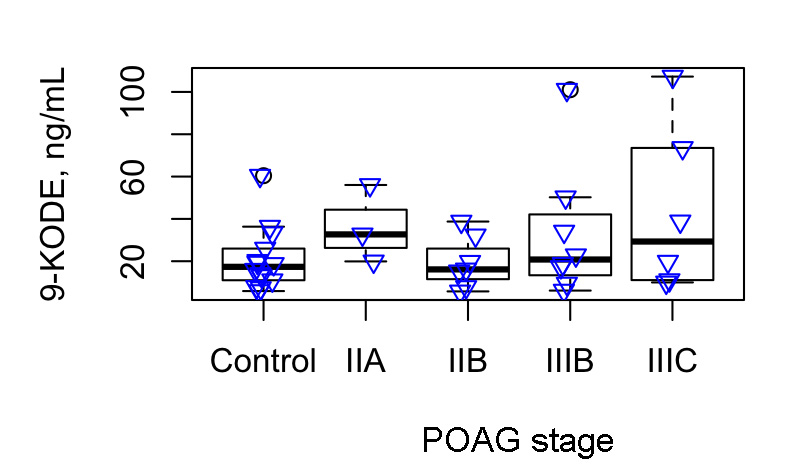

Supplement: Supplementary file 1 [file biology-10-00658-s001.zip › biology-1268784-supplementary/Azbukina et al. 2021 Figure S3.jpg]

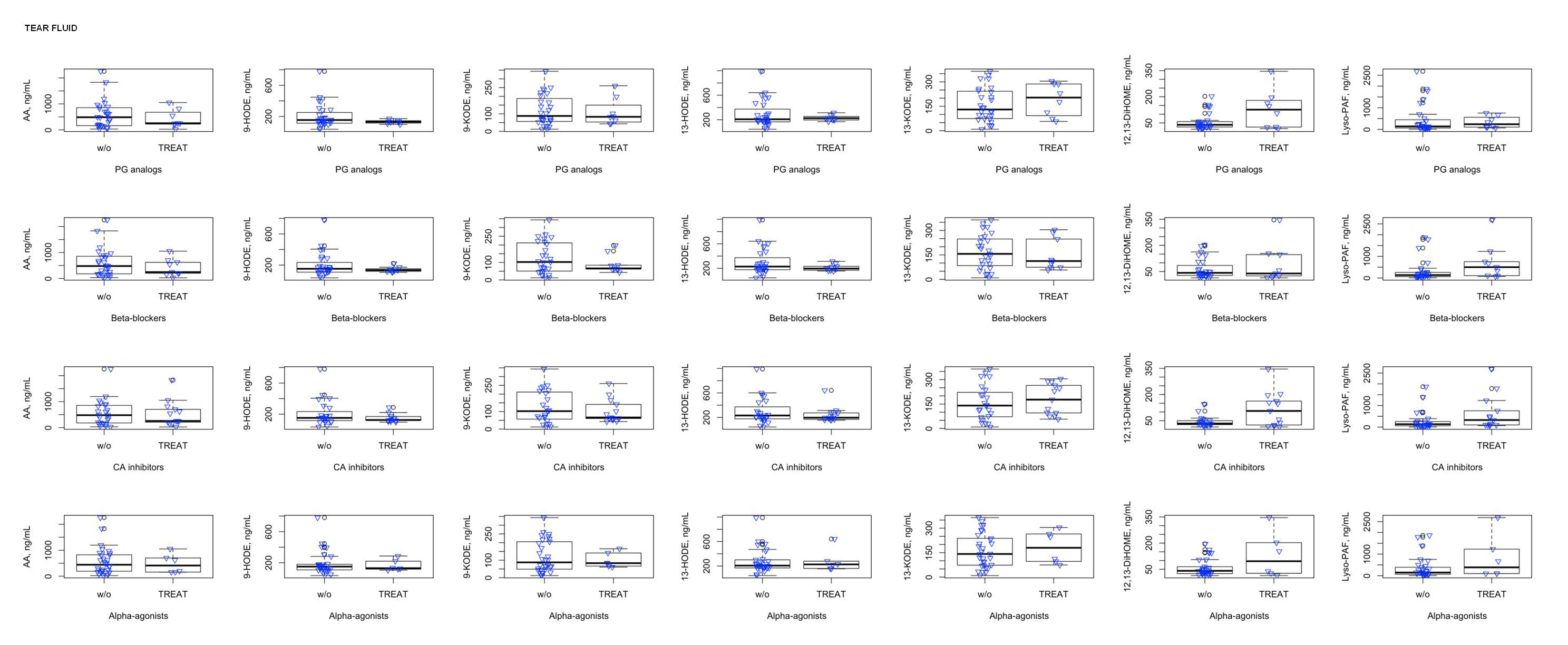

Supplement: Supplementary file 1 [file biology-10-00658-s001.zip › biology-1268784-supplementary/Azbukina et al. 2021 Figure S4.jpg]
